# European Visitor

**Published:** 1877-01

**Authors:** 


*A European Visitor.*—Dr. Leopold Peterman, of Berlin, Germany, was in Baltimore on Saturday, and visited the medical and dental colleges of the city. He expressed the opinion that the dental science in America had made greater progress toward perfection than the colleges of Europe.

